# Editorial: Contextualized Affective Interactions With Robots

**DOI:** 10.3389/fpsyg.2021.780685

**Published:** 2021-11-02

**Authors:** Myounghoon Jeon, Chung Hyuk Park, Yunkyung Kim, Andreas Riener, Martina Mara

**Affiliations:** ^1^Department of Industrial and Systems Engineering, Virginia Tech, Blacksburg, VA, United States; ^2^Department of Biomedical Engineering, George Washington University, Washington, DC, United States; ^3^iRobot (United States), Bedford, MA, United States; ^4^Faculty of Computer Science, Technische Hochschule Ingolstadt, Ingolstadt, Germany; ^5^LIT Robopsychology Lab, Johannes Kepler University of Linz, Linz, Austria

**Keywords:** affect, human-robot interaction (HRI), affective robots, affective interaction, emotions

Affect is a well-known motivating and guiding force in our daily lives. With technological advancement, there has been a growing interest to include affect in the design of complex socio-technical systems (Jeon, 2017), resulting in a new wave of applications following the embodied interaction paradigm (Marshall et al., 2013). Expressing one's own affective states and reading others' is critical for human-human interaction, to manage natural communication and social interaction. Since this is also applied to human-system interaction, researchers have started addressing affective aspects of the system in addition to cognitive aspects. However, research is still largely technology-driven, and approaches are rather general, which is often the case for the early stage of a new research area. For example, there has been much research on generic affect detection using various combinations of sensors and classification techniques (Calvo and D'Mello, 2010). But little research has focused on applying the technologies to real-world situations.

In robotics, robots have been designed for affective interactions with older adults (Smarr et al., 2014) and children with autism (Javed et al., 2019), and for hospitals (Jeong et al., 2015) and job settings (Hoque et al., 2013). Affective robots have been considered more acceptable, preferable, and trustable (Lowe et al., 2016; Bishop et al., 2019). However, there are mixed results when using affective robots (e.g., Walters et al., 2008), and more research is required to unpack the underlying mechanisms and implement the optimized interactions for different use cases.

Based on this background, this research topic invited research and design efforts that refine affective interactions with robots for specific situations and user groups. It aims to capture theories for conceptualizing affective interactions between people and robots, methods for designing and assessing them, and case studies for highlighting these interactions. We sought to elaborate on the roles of affect in contributing to a human-centered perspective that considers psychological, social, ethical, cultural, and environmental factors of implementing affective intelligence into daily human-robot interactions. The articles of this research topic included diverse contexts such as interacting with children with autism, educational setting, critical decision making, negotiation, and mixed reality. Also, the articles addressed essential constructs in affective interactions, including trust, frustration, anxiety, emotion reactions, anthropomorphism, faith, social perceptions, and copresence.

Trust formation is addressed in several pieces. Miller et al. showed that how users' trust toward a robot is formed and lasts depending on their disposition and state anxiety over time based on the distance experiment with a humanoid robot. Ullrich et al. discussed inappropriate faith in technology based on the example of a pet feeding robot. Results from their video simulation study indicate that repeated experiences with a robot as a reliable pet feeder were associated with rapidly increased trust levels and decreased numbers of control calls. Calvo-Barajas et al. adopted techniques from the Regulatory Focus Theory (Higgins, 2012) and studied the role of “promotion” and “prevention” strategies in gaining trust for HRI scenarios in educational settings. Through indirect differentiation in the behavioral expressions, the authors have embedded distinct affective impressions that resulted in changes in acceptance and trust levels. Christoforakos et al. reported two online experiments in which positive effects of robot competence and robot warmth on trust development in a humanoid robot were found, with both relationships moderated by subjective anthropomorphic attributions. In a similar line, Ullrich et al. challenged human-likeness as a design goal and questioned whether simulating human appearance and performance adequately fits into how humans build their mental models of robots and their “self.” By means of a thought experiment, the authors explored robots' attributed potential to become human-like and concluded that it might be more promising to understand robots as an “own species” to better highlight their specific characteristics and benefits, instead of designing human-like robots.

Two papers dealt with specific states. Weidemann and Rußwinkel dedicated their paper to the potential of emotional reactions, e.g., the prevention of errors or bidirectional misunderstandings, as a basis for successful human-robot interaction. In a cooperative human-robot work situation the influence of frustration on the interaction was explored. Results show clear differences in the perceived frustration in the frustration vs. the no frustration groups. Frustration also showed different behavioral interactions by the participants and a negative influence on interaction factors such as dominance and sense of control. Kim et al. explored how robot-assisted therapy may facilitate the prosocial behaviors of children with autism spectrum disorder. To this end, the authors looked at smiles, measured by annotating video-recorded behavior and by classifying facial muscle activities, and concluded that smiles indeed might be a signal of prosocial behavior.

Robots and AI influenced perceptions and decision-making procedures. Pimentel and Vinkers demonstrated that enabling a virtual human to responds to physical events in the user's environment significantly influenced users' social perception, “copresence” of the virtual human, even though there was no effect on their affective evaluation. Klichowski demonstrated the prediction by philosophers of technology (Harari, 2018), AI that people have more and more contact with is becoming a new source of information about how to behave and what decisions to make with two experiments. When the participants actually observed what AI did in which the participants had to take an urgent decision in a critical situation where they were unable to determine which action was correct, over 85% copied its senseless action. Babel et al. studied the impact of negotiation strategies in human-robot conflicts, showing that the assertive or polite negotiation skills achieved compliance from humans but negative strategies (e.g., threat, command) were less accepted.

There is no overarching framework to embrace affective interactions between people and robots, but we can postulate that it would include affect mechanisms (appraisal, reactivity, regulation, and understanding); how affective interactions can influence cognitive and behavioral processes (perception, judgment, decision-making, and action selection); and how other constructs (e.g., trust, shared situation awareness, empathy) might mediate the two. We will be able to quantify and validate these relationships based on further empirical research. This effort will help us capture the holistic relationship between people and robots and design better interactions between the two.

In sum, we hope that this research topic will provide more specific contexts in which people can develop affective interactions with robots. The combinations of these case studies will make a significant contribution to the design of affective interactions and guide us to more concrete and impactful research directions. We thank all the authors, reviewers, and editorial members for their contributions to this research topic.

## Author Contributions

The editorial was compiled by all co-editors. All authors listed have made a substantial contribution to this Research Topic and have approved this editorial for publication.

## Conflict of Interest

YK was employed by the company iRobot. The remaining authors declare that the research was conducted in the absence of any commercial or financial relationships that could be construed as a potential conflict of interest.

## Publisher's Note

All claims expressed in this article are solely those of the authors and do not necessarily represent those of their affiliated organizations, or those of the publisher, the editors and the reviewers. Any product that may be evaluated in this article, or claim that may be made by its manufacturer, is not guaranteed or endorsed by the publisher.
